# Deep‐Learning‐Assisted SICM for Enhanced Real‐Time Imaging of Nanoscale Biological Dynamics

**DOI:** 10.1002/smtd.202501080

**Published:** 2025-10-13

**Authors:** Zahra Ayar, Marcos Penedo, Barney Drake, Jialin Shi, Samuel Mendes Leitao, Igor Krawczuk, Helena Miljkovic, Aleksandra Radenovic, Jelena Ban, Volkan Cevher, Georg Ernest Fantner

**Affiliations:** ^1^ Institute of Bioengineering School of Engineering Swiss Federal Institute of Technology Lausanne (EPFL) Lausanne 1015 Switzerland; ^2^ State Key Laboratory of Robotics Shenyang Institute of Automation Chinese Academy of Sciences Shenyang 110016 P. R. China; ^3^ Institute of Electrical and Microengineering School of Engineering Swiss Federal Institute of Technology Lausanne (EPFL) Lausanne 1015 Switzerland; ^4^ Faculty of Biotechnology and Drug Development University of Rijeka Radmile Matejčić 2 Rijeka 51000 Croatia

**Keywords:** bio‐imaging, convolutional neural network, deep learning, high‐resolution, imaging, scanning ion conductance microscopy, scanning probe microscopy, SICM

## Abstract

Scanning Ion Conductance Microscopy (SICM) provides high‐resolution, nanoscale imaging of living cells, but it is generally limited by a slow scan rate, making it challenging to capture dynamic processes in real time. To tackle this challenge, an integrated data acquisition and computational framework is proposed that improves the temporal resolution of SICM by selectively skipping certain scan lines. A partial convolutional neural network (Partial‐CNN) model is developed and trained on SICM images and their corresponding masks to reconstruct the complete images from the undersampled data, ensuring the retention of structural integrity. This approach significantly reduces the image acquisition time (i.e., by 30–63%) without compromising quality, as validated through multiple quantitative metrics. Compared to conventional deep learning methods, the Partial‐CNN demonstrates higher accuracy in reconstructing fine details and maintaining consistent height maps across skipped regions. It is shown that this method provides an increased temporal resolution and retains image fidelity, making it suitable for real‐time dynamic SICM imaging and improving the smart scanning microscopy applications in time‐resolved biological imaging.

## Graphical abstract

A smart scanning algorithm combined with a partial convolutional neural network (Partial‐CNN) increases the data acquisition speed of scanning ion conductance microscopy without loss of image fidelity. The uncertainties induced by the Partial‐CNN reconstruction remain below the measurement uncertainties of two consecutive images.

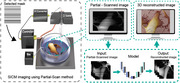

## 1 Introduction

Scanning ion conductance microscopy (SICM)^[^ ^1^ ^]^ is a subgroup of scanning probe microscopy (SPM) that is well‐recognized for capturing high‐resolution, non‐invasive topographical images of live biological samples such as cells.^[^ ^1^, ^2^, ^3^, ^4^ ^]^ In SICM, the ionic current between a nanopipette and a sample is measured and used to track the sample surface, which allows for nanoscale cellular imaging without physically contacting the sample surface.^[^ ^1^, ^5^ ^]^ High lateral resolution (on the order of tens of nanometers) and axial resolution (<5 nm),^[^ ^5^, ^6^ ^]^ combined with the ability to image in a physiological environment (ranging from simple salt solution such as phosphate‐buffered saline (PBS) to the more complex culture media such as DMEM, at 37 °C with a CO_2_ supply), make this technique highly desirable for bio‐imaging applications.^[^ ^7^, ^8^ ^]^

The non‐invasive nature of SICM offers distinct advantages over brightfield microscopy, not only in resolution but also in overcoming photosensitivity issues that can lead to phototoxicity in long‐term live imaging.^[^ ^9^ ^]^ Although atomic force microscopy (AFM)^[^ ^10^ ^]^ provides similar advantages for bio‐imaging, it induces mechanical forces on the sample, potentially triggering mechanosensitive responses in cells. SICM has proven particularly useful in imaging fine structures in living cells, such as neurons^[^ ^8^ ^]^ and stem cells,^[^ ^11^, ^12^ ^]^ without light‐induced stresses and with minimal mechanical interaction compared to techniques such as AFM. Moreover, SICM shows extensive applications in detecting nano‐mechanics^[^ ^13^, ^14^ ^]^ and measuring stiffness^[^ ^2^, ^15^ ^]^ of soft biological samples.

Despite its advantages, SICM is limited by the temporal resolution, particularly when capturing the movement of the cell body, the axial fluctuations of the cell membrane, or the dynamics of fine structures such as filopodia or growth cones. Conventional SICM setups are slow, taking minutes to capture one frame. The scanning speed in SICM depends on several factors, including the mechanical design and resonant frequency of the piezoelectric actuators, the responsiveness of the feedback system, and the efficiency of current detection and processing circuits. High‐speed SICM (HS‐SICM) addresses these limitations and can reduce imaging times to a range of seconds per image by allowing an increased hopping rate from a few Hz (e.g., 20–200 Hz)^[^ ^16^, ^17^ ^]^ up to several kHz^[^ ^3^, ^8^, ^18^, ^19^, ^20^ ^]^ on small scan sizes and flat samples. However, the required time and the distance that the probe has to move away from the surface before re‐engaging in each hop, i.e., retract time and retract height (e.g., 5–8 µm), respectively, are considerably higher for some samples like neuron cells, which reduces the effective hopping rate.

To address the SICM scanning speed limitation without requiring new equipment or hardware modifications, some previous studies focused on developing software‐based solutions to reduce the scanning time. Takahashi et al.^[^ ^8^ ^]^ developed an algorithm for the automation region of interest (AR mode) to limit the scanning area to the region with valuable data. Li et al.^[^ ^21^ ^]^ used a pre‐scanning low‐resolution technique to detect the region with valuable data to decrease the scanning time. Gu et al.^[^ ^22^ ^]^ used a target region focused (TRF) to skip the scanning of the background for an efficient scanning time. These methods are effective when there is a substantial area in the background to skip. For images in which the field of view is densely filled with cells or other biological samples, with minimal background area, these methods do not lead to a reduction in scanning time. Additionally, edge detection techniques may face challenges in accurately predicting cell positions in the following frames during live imaging due to cell movement.

Emerging technologies like deep learning, specifically convolutional neural network (CNN),^[^ ^23^ ^]^ provide advancements in the performance of microscopy techniques, including segmentation,^[^ ^24^ ^]^ super‐resolution techniques,^[^ ^25^ ^]^ and denoising.^[^ ^26^ ^]^ Similarly, Liu et al.^[^ ^25^ ^]^ and Wu et al.^[^ ^27^ ^]^ used a deep‐learning approach in AFM and SICM, respectively, to enhance the resolution and generate high‐resolution images from low‐resolution inputs. These techniques allow scanning in a lower resolution (e.g., 32 × 32 pixels) and improve the image resolution (e.g., to 256 × 256 pixels) by an autoencoder, achieving results comparable to experimentally obtained high‐resolution images. Although these models are effective, they miss fine features that are lost during the low‐resolution scan, which prevents the model from reconstructing these features.

This study introduces an alternative approach for data acquisition, which enhances imaging speed and reduces scanning time in SICM. The proposed technique selectively skips certain scan lines according to a predefined masking pattern, allowing a trained deep‐learning model to reconstruct the missing data. We use two distinct models, convolutional neural networks (CNN)^[^ ^23^ ^]^ and partial convolutional neural networks (Partial‐CNN),^[^ ^28^ ^]^ to demonstrate their effectiveness in reconstructing and inpainting images with skipped lines. We evaluate the performance of these models in reconstructing masked SICM images through visual inspection and quantitative metrics while comparing them with traditional linear interpolation methods. With this work, we contribute to developing smart microscopy techniques for scanning probe microscopy.

## 2 Results

### 2.1 Workflow of the Partial‐Scan Method

**Figure 1:**
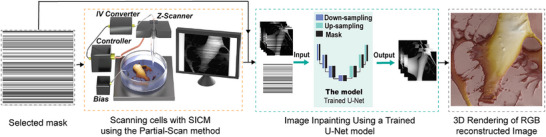
The workflow of the Partial‐Scan method in SICM. After selecting the mask, the imaging was conducted according to the white lines (scanned lines). Both images and masks were fed to a pre‐trained model to reconstruct the skipped lines. The image was visualized using a 3D RGB rendering to enhance the visual inspection.

**Figure** **1** shows the overview of the workflow for the Partial‐Scan method in SICM. A predefined mask determines which lines will be scanned and which will be skipped during the SICM partial scanning (Figure S1, Supporting Information). The images and their corresponding masks are then fed to the model trained using a collection of previously recorded SICM images to reconstruct the skipped lines. Feeding the mask alongside the data improves accuracy by focusing the computation on the unmasked regions while also making the model lighter and more efficient for the inpainting purposes of small datasets. The final reconstructed images are rendered to 3D RGB images to provide an intuitive visual representation.

### 2.2 Model Performance

Two models were trained based on CNN and Partial‐CNN using SICM images to compare their abilities to reconstruct skipped lines (Figure S2, Supporting Information). The Partial‐CNN model has significantly fewer parameters to train due to the partial convolution in its layers, making it a less complex and more computationally efficient model. In contrast, the CNN model requires additional steps to reach optimal performance, while the Partial‐CNN achieved an optimal reconstruction more quickly and is less prone to overfitting on a limited SICM data set (Table S1, Supporting Information, *p* < 0.001). We used a linear interpolation method to provide a minimum baseline reconstruction for model comparisons (Figure S3, Supporting Information)

To evaluate the quality of the reconstructions, we first acquired fully scanned images and simulated the Partial‐Scan method by removing the masked lines. This approach allowed us to compare the different reconstruction methods, linear interpolation, CNN, and Partial‐CNN, against the original data, providing a clear benchmark for assessing their performance.

**Figure 2:**
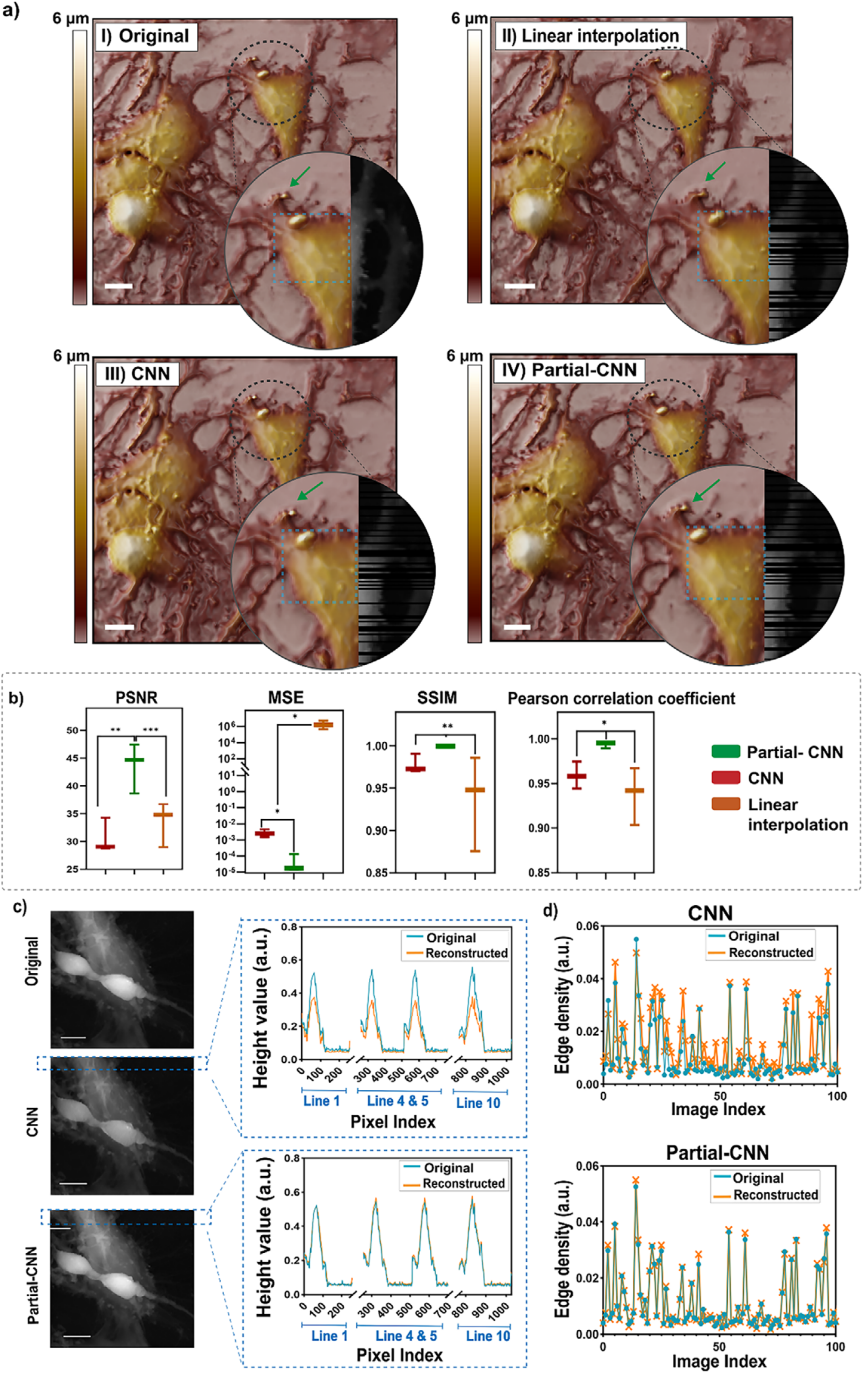
Comparison of reconstruction methods for SICM images (256 × 256 pixels) with 30% line masking; a) Visual comparison of (I) the original image and reconstructed image using (II) linear interpolation, (III) CNN, and (IV) Partial‐CNN models. Linear interpolation lacks resolution, CNN misses capturing some features (green arrows), and creates incorrect height values (inside the blue rectangle). Scale bar = 10 µm. b) Statistical metrics (PSNR, MSE, SSIM, Pearson's correlation coefficient) demonstrate a significant difference (mean ± SD, *n* = 3, *p* < 0.05) between CNN and Partial‐CNN reconstructions, supporting previous observations. c) Visual inspection and height value analysis confirm that CNN struggles to maintain fidelity to the original image height values; however, Partial‐CNN aligns closely with the original height values (scale bar = 10 µm); d) The canny method was used to calculate the edge density (number of pixels containing an edge normalized by the total number of pixels) for 100 images reconstructed using CNN and Partial‐CNN. The result shows that CNN does not preserve the edges, and the method fails to maintain edge clarity, while in Partial‐CNN, it preserves all edges effectively.

**Figure** **2**a illustrates the visual differences in reconstruction quality between linear interpolation, CNN, and Partial‐CNN, highlighting the strengths and weaknesses of each approach in preserving the image details and structure. The figure demonstrates that linear interpolation cannot faithfully preserve unscanned details, especially in regions with fine structures. The CNN‐based reconstruction shows overall high quality and can capture most of the fine features, but some elements are still lost in areas with intricate details (green arrows). Comparing the CNN reconstruction with the Partial‐CNN result shows that the Partial‐CNN provides higher accuracy in capturing small details and fine elements (green arrows). In the region marked by the blue dashed rectangle, the CNN model occasionally fails to estimate the correct height for missing areas and produces an incorrect height value in the reconstruction.

The statistical analysis between CNN, Partial‐CNN, and linear interpolation, shown in Figure 2b, supports the visual observations, showing that the Partial‐CNN performs better in image reconstruction compared to both other models. Metrics such as Peak Signal‐to‐Noise Ratio (PSNR), Mean Squared Error (MSE), Structural Similarity Index Measurement (SSIM), and Pearson's correlation coefficient reveal statistically significant benefits of the Partial‐CNN (*p* < 0.05). Although the CNN and linear interpolation performance in PSNR, SSIM, and Pearson's correlation coefficient does not show significant performance, the MSE has a significant difference, showing that linear interpolation causes pixel‐wise errors that lead to blurriness of the reconstructed image.

The reconstructed images in Figure 2.c reveal that the CNN reconstruction cannot estimate the height values for the lost region compared to the Partial‐CNN reconstruction. The height value when using Partial‐CNN matches the original image, while the height value in the reconstructed lines is underestimated when using CNN. More investigation using the Canny method is performed to confirm the preservation of the edges. The calculation of the edge density of 100 images reconstructed by Partial‐CNN and CNN is shown in Figure 2.d, which confirms lower preserved edges in images reconstructed using the CNN model compared to Partial‐CNN.

### 2.3 Real‐Life Image Acquisition Using the Partial‐Scan Method

**Figure 3:**
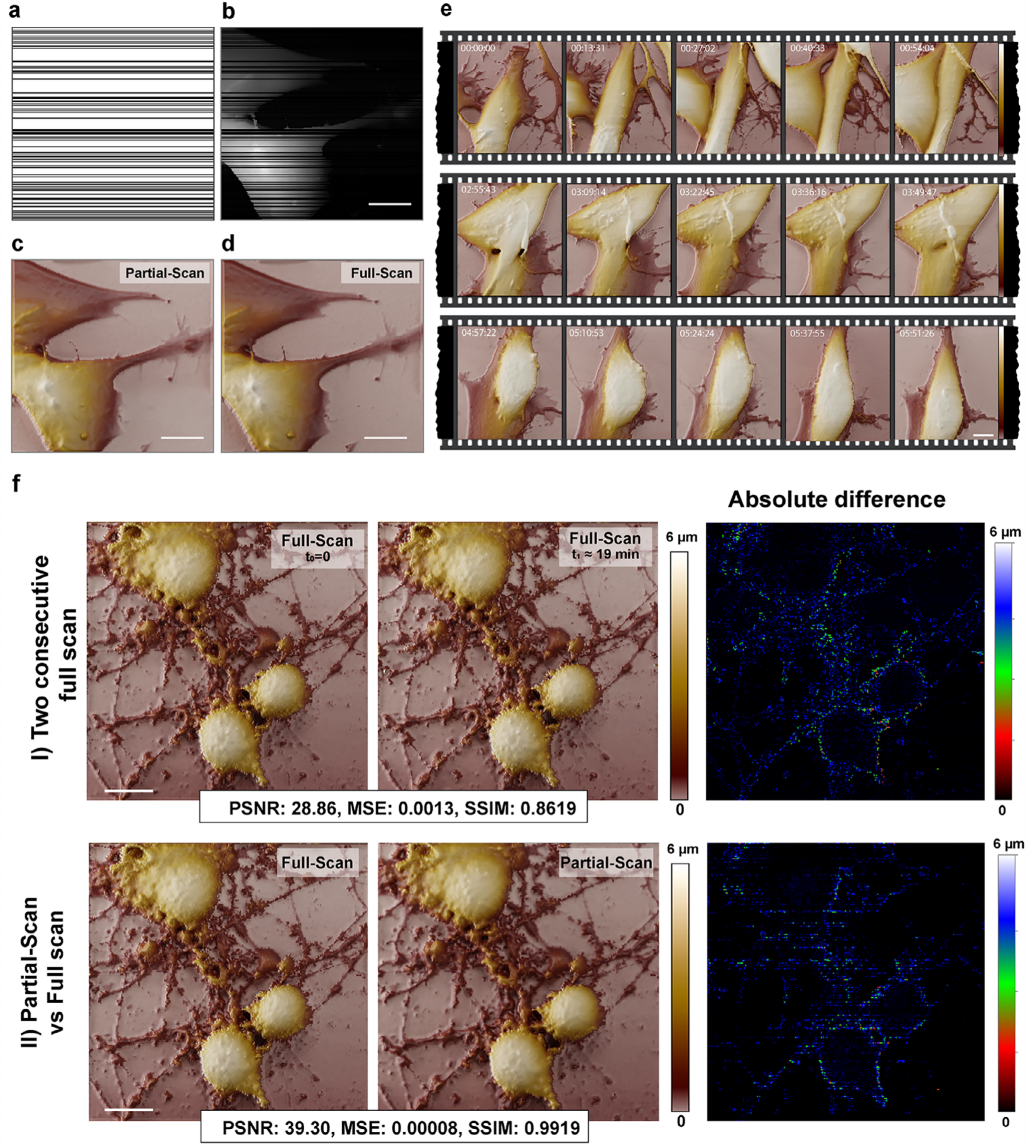
Evaluation of the Partial‐Scan method and image reconstruction in SICM on fixed and live cells using Partial‐CNN; a) mask for skipped‐line imaging (30% masking), b) partial scanned image, c) reconstructed image using the Partial‐CNN model, d) the full‐scan image for reference, used to assess reconstruction accuracy. e) Time‐lapse sections of live‐imaging of neuroblastoma cells for >9 h using the skipped line method. The Partial‐Scan using Partial‐CNN reduces the time from 19 min and 19 s to 13 min and 31 s. f) Comparison of the absolute difference in I) two consecutive full‐scan images in SICM, and II) using the Partial‐Scan method on fixed cells, shows that the uncertainty in the reconstruction based on Partial‐CNN is lower than the inherent uncertainty of the instrument; Scale bars = 10 µm.

The Partial‐Scan method reduces the image acquisition time by reducing the number of scanned lines based on a predefined mask (e.g., 30% masking, **Figure** **3**a), where lines within the white regions of the mask are scanned, while black lines are skipped (Figure 3b). To validate this approach, we first imaged the fixed SH‐SY5Y human neuroblastoma cells using the Partial‐Scan method and then imaged the same region using a full‐scan method. The comparison between the reconstructed images using the Partial‐CNN model (Figure 3c) and the original full‐scan image (Figure 3d) highlights the high efficacy and accuracy of the model in reconstructing the skipped lines (PSNR: 43, MSE: 1.5 ×10^−4^, SSIM: 0.99).

After validating the method on fixed samples, we applied it for imaging live SH‐SY5Y neuroblastoma cells. The live cells were maintained in a custom‐built incubator at 37 °C with a controlled CO_2_ supply for over 9 h. Figure 3e presents sections from the time‐lapse imaging of live cells, presenting the success of the Partial‐CNN model in reconstructing skipped lines over extended imaging sessions. The Partial‐Scan method reduces scanning time from 19 min 19 s to 13 min 31 s, achieving a 30% reduction. This time savings is critical for live‐cell imaging, allowing for capturing more frames over time and enabling the observation of faster cellular movements and dynamics with greater accuracy (Video S1, Supporting Information).

Figure 3f shows that consecutive full‐scan images of the same area in the fixed sample differ due to environmental factors, artifacts, or system noise. Compared to two consecutive full‐scans (PSNR: 28.86, MSE: 0.0013, SSIM: 0.8619), the Partial‐Scan reconstruction achieves higher fidelity (PSNR: 39.30, MSE: 8 × 10 −5, SSIM: 0.9919). This indicates that the uncertainty of the model is lower than the inherent uncertainty of the SICM instrument.

### 2.4 Model's Break‐Point and Masking Effect

**Figure 4:**
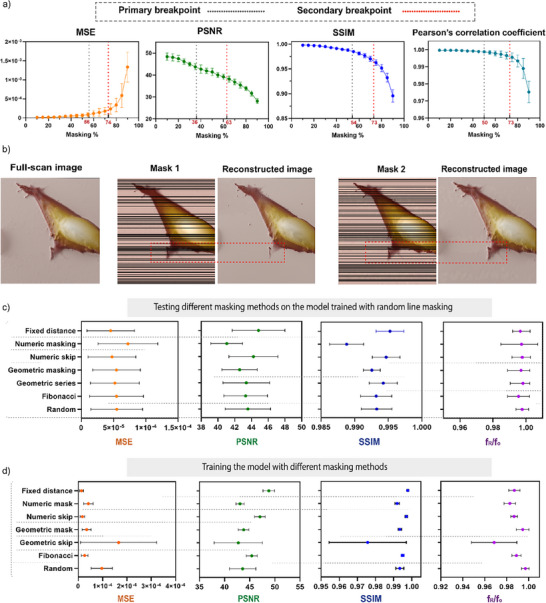
Model evaluations: a) Breaking point assessments show the primary break‐point ≈36% with the highest reconstruction potential according to all metrics. The metrics show acceptable reconstruction performance until 63%, with the possibility of some artifacts (mean ± SD, *n* = 3). b) Examples of the controlled‐pattern masking methods generated using various logical patterns to assess the effect of masking on model performance, c) Evaluation of the potential of using different types of masks in the Partial‐CNN model trained by random masking using the evaluation of PSNR, MSE, SSIM, and frequency content, the ratio of f_R_, the frequency content of the reconstructed image, and f_O_, the frequency content of the original image. The result shows no significant difference (mean ± SEM, *n* = 3). d) Performance comparison of the Partial‐CNN models trained using different masking methods based on PSNR, MSE, SSIM, and frequency content (mean ± SEM, *n* = 3). Although all the masking methods, except the Geometric mask, show no significant difference in PSNR, MSE, and SSIM, they all show lower frequency content compared to the random selection, which shows the effectiveness of the Random mask for training.

The performance evaluation of the Partial‐CNN model across different masking percentages using PSNR, MSE, SSIM, and Pearson correlation coefficient in **Figure** **4**a reveals break points for the models calculated using segmented regression. Analysis of all metrics reveals that up to the primary break‐point of 36% masking, the model performs well, maintaining high reconstruction quality with minimal artifacts and strong metrics across all evaluations. Between the primary break‐point (36%) and the secondary break‐point (63%), the model shows acceptable reconstruction with a gradual decline in all metrics, indicating challenges in accurately reconstructing fine details and maintaining structural integrity. Beyond 63% masking, the performance significantly deteriorated, with substantial loss of details and high‐frequency information, making reconstruction unreliable (Figure S4, Supporting Information).

However, uncontrolled random masking could reduce the reconstruction quality if excessive information is lost in specific regions (Figure 4b). To avoid data loss, we can limit the number of consecutive masked lines or train the model based on a controlled pattern mask, especially in higher masking percentages.

To assess if the masking pattern affects the image reconstruction quality, we generated several controlled masking patterns (Figure S5, Supporting Information), each generated based on a distinct logical approach (see Table S2, Supporting Information). Statistical analysis between the metrics in Figure 4c reveals that using the different types of masking for a Partial‐CNN model trained by the Random mask shows no significant difference in the model performance, indicating the flexibility of this model for using any mask. We also evaluated whether the model trained using different controlled masking methods can provide an advantage over the Random line selection (Figure 4d). Although the performance in PSNR, MSE, and SSIM shows no significant difference, except for the Geometric mask, the frequency content, which is the ratio of f_R_ (the frequency content of the reconstructed image), and f_O_ (the frequency content of the original image), reduces in all the other masking methods compared to the Random mask. The results indicate that training the model with a specific pattern (e.g., Fixed distance) can lead to the loss of information in the frequency domain due to the fixed and repetitive pattern.

### 2.5 Using Partial‐CNN to Remove Inherent Scan Artifacts

**Figure 5:**
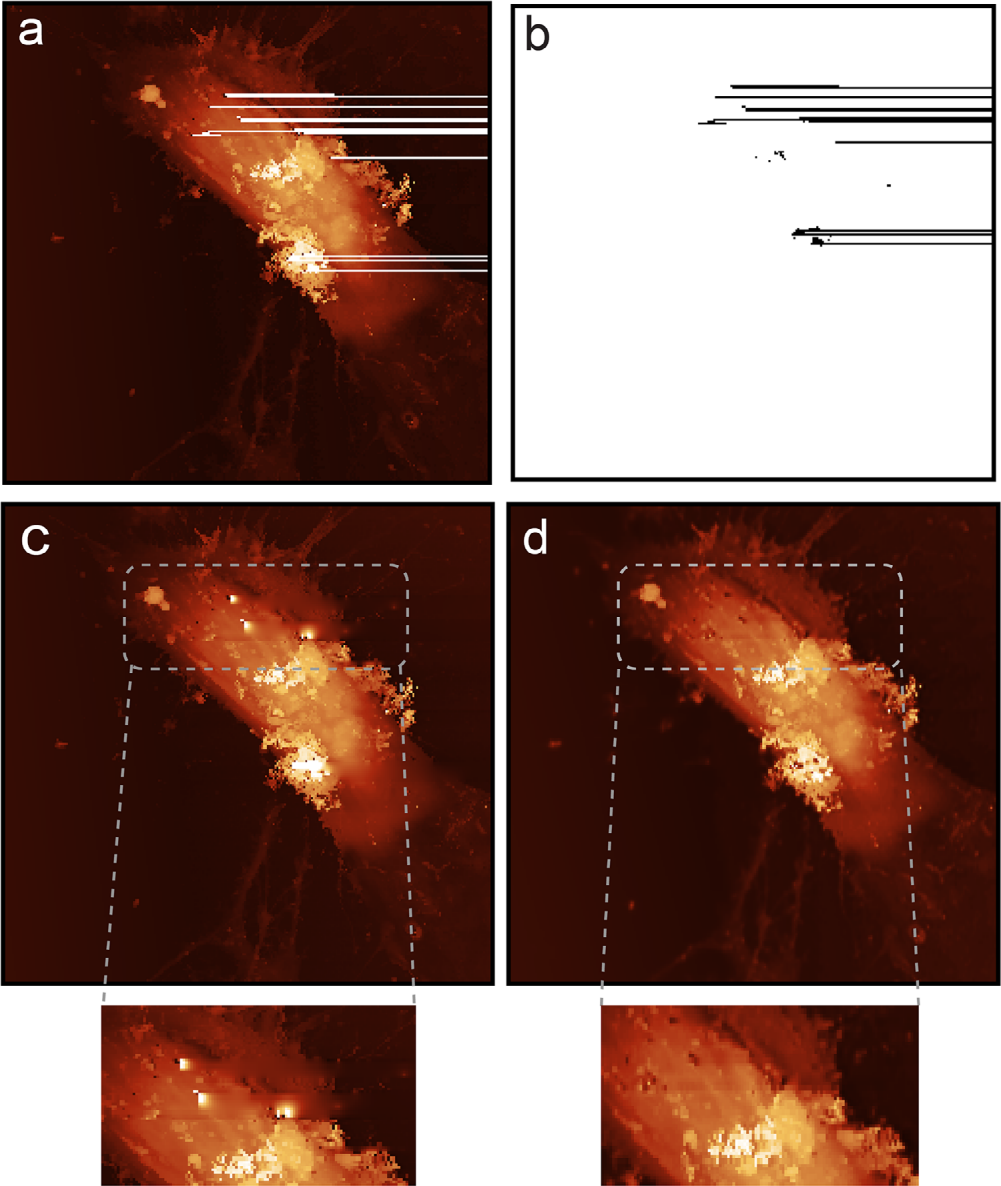
The extended application of the Partial‐CNN model in scar correction: a) scarred image, b) extracted mask correlated with the scar pattern, c) reconstructed lines using the median of lines method (Gwyddion software), d) reconstructed lines using the Partial‐CNN model.

Beyond improving scanning speed and saving time in SICM, Partial‐CNN models address additional challenges in SICM and, more broadly, scanning probe microscopies (SPM). Issues such as a contaminated medium or pipette overshooting from feedback delays can introduce artifacts, or “scars,” into the image, affecting its quality and usability. Traditional techniques like median line correction are often used to address these scars. Tools such as the open‐source SPM image processing software Gwyddion use median‐based methods to smooth out line defects by averaging neighboring lines, which can be effective for minor artifacts. However, as shown in **Figure** **5**a, median‐based interpolation struggles to reconstruct these areas accurately when scarring is extensive. By considering the scarred regions as a mask (Figure 5b), the Partial‐CNN model effectively predicts and reconstructs the missing information (Figure 5d) without introducing the blurriness often seen with median‐based methods. The comparison between median line correction and Partial‐CNN reconstruction highlights the Partial‐CNN model's advantage in preserving image clarity, showing its broader potential for image processing. Moreover, here we demonstrated the model's successful application to a completely unseen cell type (i.e., astrocytes), further confirming its robust generalization to reconstruct previously unseen data.

## 3 Discussion

The scanning rate is a critical factor in scanning probe microscopy, especially in studies of dynamic phenomena in biological samples. In AFM, scanning rates have advanced rapidly due to hardware improvements and feedback control optimization, reaching an imaging rate of several Hz.^[^ ^29^, ^30^, ^31^, ^32^ ^]^ Alongside hardware developments, software‐based solutions have explored subsampling methods for AFM to reduce imaging time.^[^ ^8^, ^22^, ^27^ ^]^ Traditional compressed sensing methods^[^ ^33^, ^34^, ^35^ ^]^ have been employed successfully for subsampling and reconstructing AFM images, though they are limited by their reliance on mathematical models and a constrained number of parameters for optimization. However, deep learning allows us to train models with millions of parameters quickly, providing high performance in image reconstruction. However, deep learning reduces the control over model behavior due to the “black‐box” nature of neural networks.

While traditional models have limitations in achieving higher reconstruction metrics, not all deep learning methods consistently reach the accuracy required for reliable imaging. The comparison of PSNR and other metrics in previous studies^[^ ^33^, ^34^, ^35^ ^]^ highlights the significant difference between the traditional compressed sensing methods and the current application of Partial‐CNN in the reconstruction of the skipped lines. Earlier methods based solely on compressed sensing, such as those presented by Han et al.^[^ ^34^ ^]^ and Luo & Andersson,^[^ ^35^ ^]^ typically achieved PSNR values ranging between 28 and 32 dB (with no SSIM value reported), indicating limited reconstruction fidelity. More recent models like ISTA‐Net^[^ ^36^ ^]^ have improved these results, reporting PSNR values ≈31.5 dB and SSIM of 0.88. However, the Partial‐CNN outperforms these approaches, achieving a PSNR of 43 dB and SSIM of 0.99 on the SICM dataset. Also, analysis of metrics (i.e., PSNR, MSE, SSIM, Pearson's correlation coefficient), and height map confirm that Partial‐CNN also outperforms traditional CNN in this application. As shown in Figure 2, CNNs^[^ ^37^ ^]^ struggle with preserving the details, often introducing height value mismatches compared to the original images, while Partial‐CNN^[^ ^28^ ^]^ preserves these fine details more effectively. This improvement is attributed to using the mask during training for Partial‐CNN, which reduces the trainable parameters and focuses on the relevant image regions.

A key factor contributing to this improvement is the nature of the training data. Previous studies, such as that by Luo et al.,^[^ ^38^ ^]^ which combined U‐Net and RED‐Net architectures, were trained on artificial datasets (e.g., animal images). In contrast, our Partial‐CNN was trained directly on real SICM data acquired from cellular samples. This likely explains the performance gap, with their model achieving PSNR values in the range of 32–35 dB and SSIM ≈0.91, compared to 43 dB and 0.99 SSIM achieved by our Partial‐CNN approach. Moreover, thanks to the presence of the binary mask alongside the input images, our Partial‐CNN model is optimized for efficient training with a relatively small dataset^[^ ^28^ ^]^ (<1000 training images), which is significantly smaller than the datasets used in other models.^[^ ^36^, ^39^ ^]^

To provide a more comprehensive evaluation of the model's capabilities, we also assessed edge preservation, model breakdown thresholds, and the impact of different masking strategies on model performance. Our analysis of the model's break‐point in Figure 4a shows that the optimal masking level is ≈30–36%, offering a balance between increased temporal resolution and preserved image structure. Further tests confirmed that this masking level could be reliably increased to 63% if a small loss in image fidelity is acceptable. Training the model with random masks is recommended to retain flexibility when choosing varied line‐skipping patterns. As also shown in Figure 4, careful selection of the mask is crucial to avoid excessive masking in specific regions, which can cause significant information loss and compromise reconstruction quality.

Unlike many previous studies^[^ ^33^, ^34^, ^35^, ^36^ ^]^ that remained limited to the simulated under‐sampled data derived from full‐scan images, we aimed to validate our approach directly on SICM measurements to ensure accurate real‐world performance. As shown in Figure 3, our study demonstrates that the Partial‐Scan method (with the Partial‐CNN model) effectively reduces SICM imaging time for both fixed and live cells by selectively masking scan lines while preserving the high reconstruction metrics. The required imaging time depends on the biological target. While fixed or static samples can be scanned in 10–20 min, dynamic live‐cell processes such as membrane ruffling^[^ ^40^ ^]^ and filopodia activity^[^ ^41^ ^]^ typically require a resolution of 1–5 min. In our earlier work, we achieved this speed in flat cells like kidney and melanoma cells by using a low retract height (i.e., 1–2 µm).^[^ ^18^ ^]^ However, imaging complex morphologies, such as neurons with elevated soma regions, demands higher retract heights (i.e., 5–8 µm) and longer scan times. In this study, we reduced scan time from 19 min and 19 s to 13 min and 31 s using Partial‐CNN with 30 percent masking while maintaining high fidelity. By increasing the masking rate to 60 percent and using a retract height of 5–8 µm, we can reduce the scan time to ≈5 min. Although higher masking introduces some compromises, the model preserved essential structural features with acceptable fidelity, as shown in Figure S4 (Supporting Information). These results highlight the potential of our Partial‐CNN approach to empower SICM imaging even for challenging cell types such as neurons.

The evaluation between two images in a consecutive full scan of a fixed sample in Figure 3 shows that there are naturally minor differences between the two images in different consecutive scans due to the presence of artifacts in the media, noise, and other environmental factors. The results in Figure 3f show that the Partial‐Scan is a robust and reliable method, showing lower absolute differences and higher performance (PSNR: 39.30, MSE: 8 × 10 ^−5^, SSIM: 0.9919) compared to the natural variations between two consecutive images in SICM (PSNR: 28.86, MSE: 0.0013, SSIM: 0.8619), demonstrating its robustness and reliability under practical imaging conditions. Figure S6 (Supporting Information) also shows that the difference between the two partially scanned images is not significant. The low value of MSE in two consecutive Partial‐Scan indicates that the method metrics are stable over different scans.

Nevertheless, while the Partial‐CNN outperformed a standard CNN and traditional models in preserving boundary structures, particularly at cell‐substrate interfaces, we observed that reconstruction fidelity decreases slightly in regions with pronounced topographic changes, such as steep edges around neuronal soma (shown in Figure 2c,d). This limitation is partly due to reduced height cues in masked data and inherent SICM scanning constraints in high‐gradient areas. Further improvements in future studies may include edge‐aware loss functions for more accurate training in the edge area, or post‐processing approaches such as gradient‐based sharpening filters to enhance boundary reproduction under challenging topographies.

Furthermore, the application of the Partial‐CNN model for scar correction, shown in Figure 5 on an unseen cell type (i.e., astrocytes), highlights the generalization capability of the model. Recent comparative studies on state‐of‐the‐art denoising and scar correction, such as Restormer, HINet, and Uformer^[^ ^39^ ^]^ show lower performance, with PSNR values below 28.5 dB and SSIM ≈0.83–0.85 in the presence of line and scar noise. In addition to their low performance metrics, these models operate only on full‐image inputs and are not designed to account for sampling masks.

While this method demonstrates high reconstruction accuracy, certain limitations must be considered. While the comparisons verify the fidelity of partial‐CNN reconstructions, minor height value misestimations may occur. We advise that images will be labeled as AI‐enhanced reconstructions. It should also be closely monitored that skipping lines does not create a stepped pattern in the edges of the reconstructed images.

Future work can combine the Partial‐Scan method with previous software solutions^[^ ^8^, ^22^ ^]^ for background skipping,^[^ ^8^ ^]^ which could further reduce scanning time by omitting background areas in each line and applying the Partial‐Scan method only to regions of interest. Furthermore, with appropriate data, the existing trained model could serve as a strong foundation for a transfer learning approach, significantly accelerating the training of models for predicting charge or mechanical properties.

## 4 Conclusion

This study successfully improved the temporal resolution of SICM imaging by implementing a Partial‐Scan method with a deep learning‐based model trained to reconstruct the skipped lines. This technique applied advanced methods to develop Smart‐SICM imaging as a subgroup of smart microscopy techniques in SPM. Utilizing a partial convolutional neural network (Partial‐CNN) resulted in superior performance to traditional CNNs, achieving more accurate reconstructions with preserved detail. The time saved was directly related to the percentage of skipped lines, with 30% masking identified as an optimal balance, significantly enhancing temporal resolution while maintaining the structural fidelity of the sample, making it desirable for real‐time live imaging of cells.

## 5 Experimental Section

### Image Acquisition Using Scanning Ion Conductance Microscopy

A custom‐built SICM setup was established and used for imaging.^[^ ^18^ ^]^ The system consists of a custom Z‐actuator driven by a home‐built controller for feedback control of fine movements and a stepper motor for coarse movement. The Z‐actuator covers a high range of scanning in Z with a maximum of 22 µm. An XY scanner (P‐517 CL Piezo NanoPositioner, Physik Instrumente) with a 100 µm travel range was used for moving the sample in X and Y, which is driven by a low‐voltage amplifier (E501 Piezo Controller System, Physik Instrumente). The XY scanner and Z actuator are assembled on an inverted microscope (Olympus IX71). A custom‐made LabVIEW software has been developed for instrument control. Nanopipettes were made using borosilicate (Sutter Instrument) with a CO_2_ laser puller (Model P‐2000, Sutter Instruments) with an inner radius of 40–60 nm. To skip the lines, a mask will be selected. Then, the modified slow scan axis signal (Y‐signal) is calculated based on the selected mask and sent to the SICM instrument to skip the determined lines. To avoid creep, the signal will be changed by increasing the Y‐axis slope instead of using step‐wise motion. Then, the sample is imaged with only the non‐masked lines acquired during the scan, reducing the scanning time.

### Cell Culture

SH‐SY5Y human neuroblastoma cells were cultured in 3.5 cm Petri dishes (ibidi, 81 156) using cell culture medium consisting of DMEM/F12 (Sigma‐Aldrich, Gibco, 11 320 033) supplemented with 10% fetal bovine serum (FBS, Sigma‐Aldrich, F7524), 1% penicillin‐streptomycin (PenStrep, Gibco, 15 140 122), and maintained in a humidified custom‐built incubator inside SICM with 5% CO_2_ at 37 °C.

### Data Preparation

A total of 815 single‐channel, greyscale SICM images (256 × 256 pixels) were obtained from various cell types, including monkey kidney fibroblast‐like cells (COS‐7), human melanoma cells (SKMEL), mouse melanoma cells (B16−F1, ATCC), human cervical cancer cells (HeLa), human neuroblastoma cells (SH‐SY5Y), and cortical neuron cells derived from opossum.^[^ ^42^ ^]^ Ten percent of the images were set aside for testing, with the remaining split into training and validation sets (80% and 20%, respectively). Initial data observations ensured no leakage between sets. Predict data sets were used for real‐life reconstruction. Due to the small dataset size, K‐fold cross‐validation^[^ ^43^ ^]^ was applied to reduce the risk of overfitting. This method divides data into three different sets (folds), with respective ratios for training, validation, and testing. Models were trained on each fold, and average metric values were reported. All trained data were normalized before training.

### Masking Methods

The images were masked using various techniques, with the masking percentage maintained between 30 and 35% of lines for model performance tests. Details of the controlled masking methods are provided in Table S2 (Supporting Information). In each method, white lines were scanned while black ones were skipped.

### Network Architecture and Implementation

Two U‐Net‐based^[^ ^44^ ^]^ models were developed to evaluate image inpainting, using consistent hyperparameters (batch size: 16, learning rate: 0.0005, alpha: 0.96) for both models. The pre‐trained weights were not used. The first model, CNN, employs an encoder‐decoder structure with filters ranging from 64 to 512, incorporating 2D convolutional layers, max‐pooling, and dropout (20%) for regularization (Figure S2, Supporting Information). The combined loss function is defined as:

(1) $$\begin{array}{ccc} L_{Combined}\;\left(y_{true}-\;y_{predict}\right) & = & \alpha .\;L_{SSIM}\left(y_{true}-\;y_{predict}\right)\\ & & +\left(1-\alpha \right)\;L_{MSE}\left(y_{true}-\;y_{predict}\right) \end{array}$$

where *L_Combined_* is the total cost of the models, *y_true_* is the original image, *y_predict_* is the predicted image, α is the learning rate, and *L_SSIM_* is

(2) $$L_{SSIM}=1-mean\left(SSIM\left(y_{true}-\;y_{predict}\right)\right)$$

Which calculates the structural similarity between two images and, *L_MSE_* is:

(3) $$L_{MSE}=\frac{1}{n}\;\sum_{i=1}^{n}{\left(y_{true}-\;y_{predict}\right)}^{2}$$

The second model, Partial‐CNN,^[^ ^28^ ^]^ shares the CNN architecture but incorporates partial convolution layers and accepts both the masked image and mask as inputs. Element‐wise multiplication before each convolution restricts processing to unmasked regions, with the mask dynamically updated during encoding and decoding. The loss function mirrors that of CNN. Models were trained on an NVIDIA A100‐SXM4‐80GB GPU. A linear interpolation technique scales images from 128 × 128 to 256 × 256 pixels as a baseline for reconstruction for visual inspections.

### Model Metrics

The model performance was evaluated using different mathematical metrics, including:

*Mean Squared Error (MSE)*: The average of the squares of the differences between the original image and the reconstructed image.

(4) $$MSE=\frac{1}{m.n}\;\sum_{i=1}^{m}\sum_{j=1}^{n}{\left(I_{original}\left(i,j\right)-\;I_{reconstructed}\left(i,j\right)\right)}^{2}$$

where m and n are the dimensions of the image, and I is the pixel value in coordination with i and j.

*Peak Signal‐to‐Noise Ratio (PSNR)*: To evaluate image reconstruction quality.

(5) $$\mathrm{PSNR}=10.\;Log\;(\frac{{\mathrm{MAX}}_{I}^{2}}{\mathrm{MSE}})$$

where MAX_I_ is the maximum possible pixel value of the image, and MSE is the mean squared error between the original and reconstructed images.

*Structural Similarity Index Measure (SSIM)*: To assess the perceptual similarity between two images based on the structural information, contrast, and luminance between the original and reconstructed images.

(6) $$SSIM\;\left(x,y\right)=\frac{\left(2\mu_{x}\mu_{y}+C_{1}\right)\left(2\sigma_{xy}+C_{2}\right)}{\left({\mu_{x}}^{2}{\mu_{y}}^{2}+C_{1}\right)\left({\sigma_{x}}^{2}+{\sigma_{y}}^{2}+C_{1}\right)}$$

where x is the original image, and y is the reconstructed image, μ_*x*_ and μ_*y*_ are the average (mean) value of x and y, σ_*x*_ and σ_*y*_ are the variances of the original and reconstructed images, and *C*_1_ and *C*_2_ are constants.

*Edge density measurement*: The Canny edge detection method^[^ ^45^ ^]^ was used to detect the ratio of the edge pixels to the total pixel size in 100 images to determine the efficacy of the model in preserving the edges in the reconstructed image.

### Statistical Analysis

The preprocessing of images was handled using Gwyddion software. Skimage and Numpy Python libraries were used to calculate metrics (i.e., PSNR, MSE, SSIM, edge density, *n* = 3). For statistical analysis, images have been aligned to eliminate any mismatch caused by drift. All data were analyzed using GraphPad Prism. One‐way ANOVA was performed to compare groups (*n* = 3), followed by Tukey's multiple comparison test. For two‐group comparisons, unpaired t‐tests were used. In these comparisons, *p* < 0.05 was considered statistically significant. Metrics have been reported as mean ± SD (*n* = 3), and the effect of masking method reported based on mean ± SEM (*n* = 3). The 3D reconstruction of images was done using ImageJ and Blender 4.1 software. Model breakdown points are calculated based on segmented linear regression (Segment = 3).

## Conflict of Interest

The authors declare no conflict of interest.

## Supporting information



Supporting Information

Supplemental Video 1

## Data Availability

The data that support the findings of this study are available from the corresponding author upon reasonable request.
