# Supplementary material for: Deep‐Learning‐Assisted SICM for Enhanced Real‐Time Imaging of Nanoscale Biological Dynamics
Source: Small Methods. 2025 Oct 13;9(12):e01080. doi: 10.1002/smtd.202501080 (PMC12716206; doi:10.1002/smtd.202501080)
Supplement: Supplementary file 1 — Supporting Information [file SMTD-9-e01080-s001.docx]

**Supporting Information**

Deep-Learning-Assisted SICM for Enhanced Real-Time Imaging of Nanoscale Biological Dynamics

Z. Ayar^1^, M. Penedo^1^, B. Drake^1^, J. Shi^1,2^, S. M. Leitao^1^, I. Krawczuk^2^, H. Miljkovic^1^, A. Radenovic^1^, J. Ban^3^, V. Cevher^2^, and G. E. Fantner^1,*^

Affiliation 1: Z. Ayar, M. Penedo, B.Drake, J.Shi, S.M. Leitao, H.Miljkovic, A. Radenovic, G.E. Fantner: Institute of Bioengineering, School of Engineering, Swiss Federal Institute of Technology Lausanne (EPFL), Lausanne 1015, Switzerland

Affiliation 2: J. Shi: State Key Laboratory of Robotics, Shenyang Institute of Automation, Chinese Academy of Sciences, Shenyang 110016, PR China;

Affiliation 3: I. Krawczuk, V. Cevher: Institute of Electrical and Microengineering, School of Engineering, Swiss Federal Institute of Technology Lausanne (EPFL), Lausanne 1015, Switzerland

Affiliation 4: J. Ban: Faculty of Biotechnology and Drug Development, University of Rijeka, Radmile Matejčić 2, 51000 Rijeka, Croatia

Corresponding Author: Georg.fantner@epfl.ch


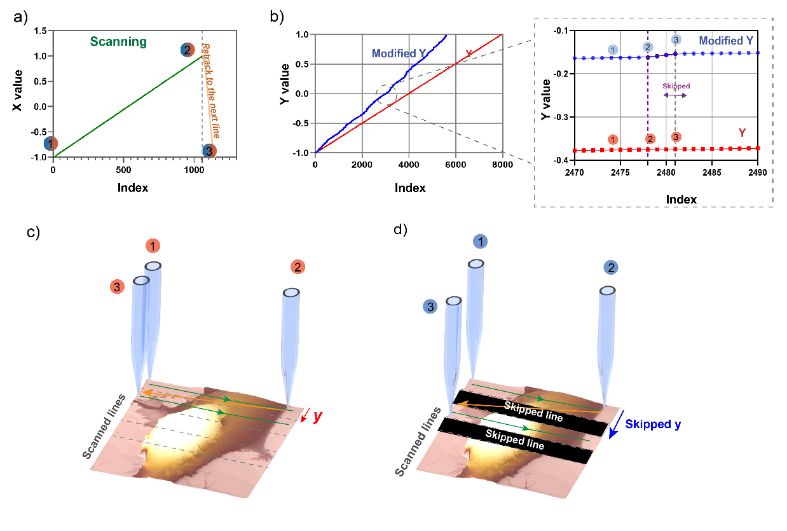


**Figure S1**. The movements of x and y are precisely controlled by the XY scanner stage and its controller. Scanning in the x direction begins at point one (pixel 1) and proceeds to point 2 (the last pixel, e.g., 256). The stage then returns to the beginning of the next line at point 3. Typically, the y-axis moves at a constant linear speed while the x-axis returns from point 2 to point 3. However, in the skip-line method, the y-axis movement follows the selected mask, resulting in a steeper y-axis slope. This allows for skipping more lines (e.g.,2y) within the same time frame that the x-axis returns to the following scanned line (from point 2 to point 3). This steeper movement reduces the overall scanning time without compromising the acquired resolution.


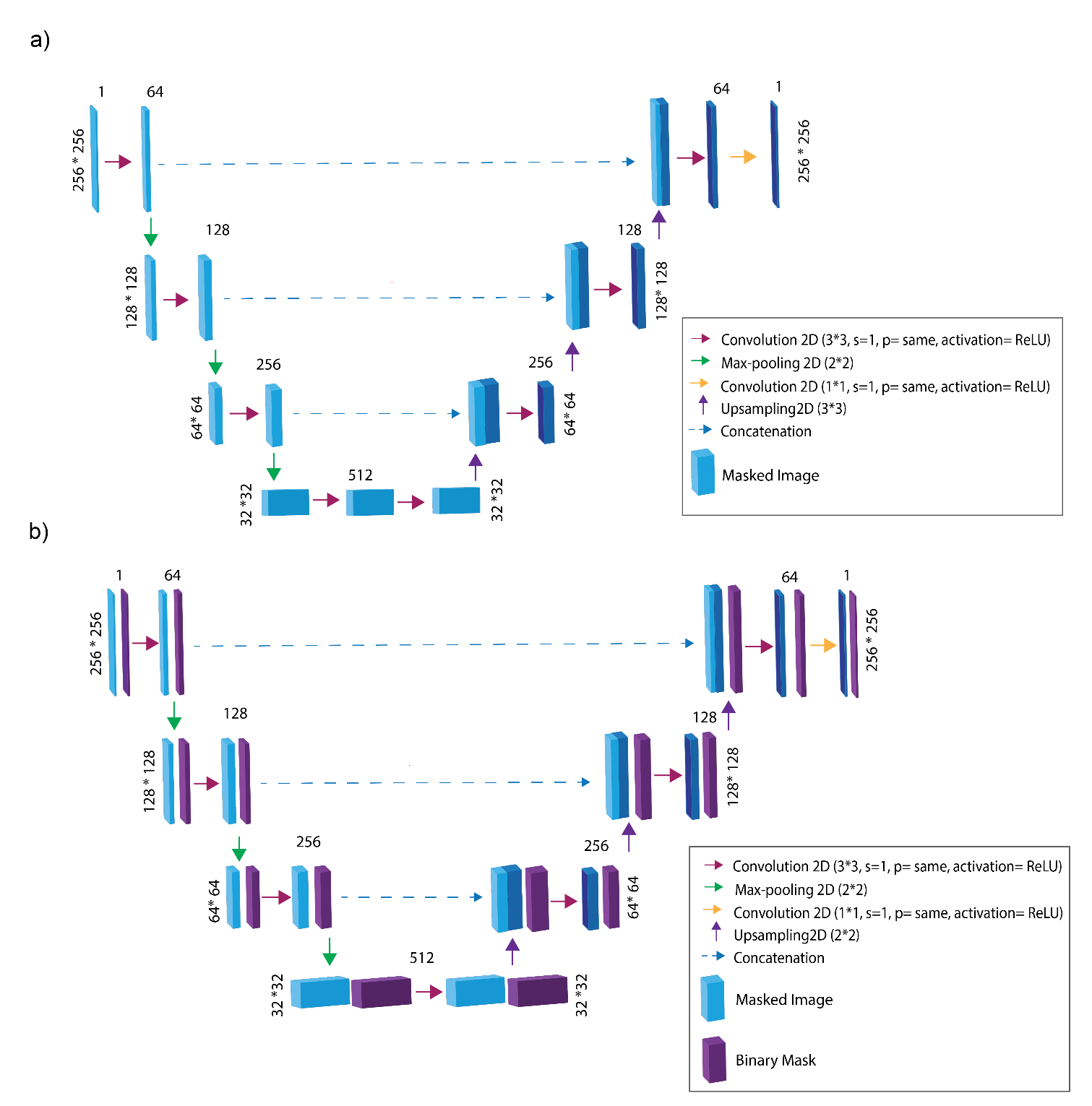


**Figure S2**. The schematic of neural network models. a) Convolutional neural network (CNN), b) partial convolutional neural network (Partial-CNN)


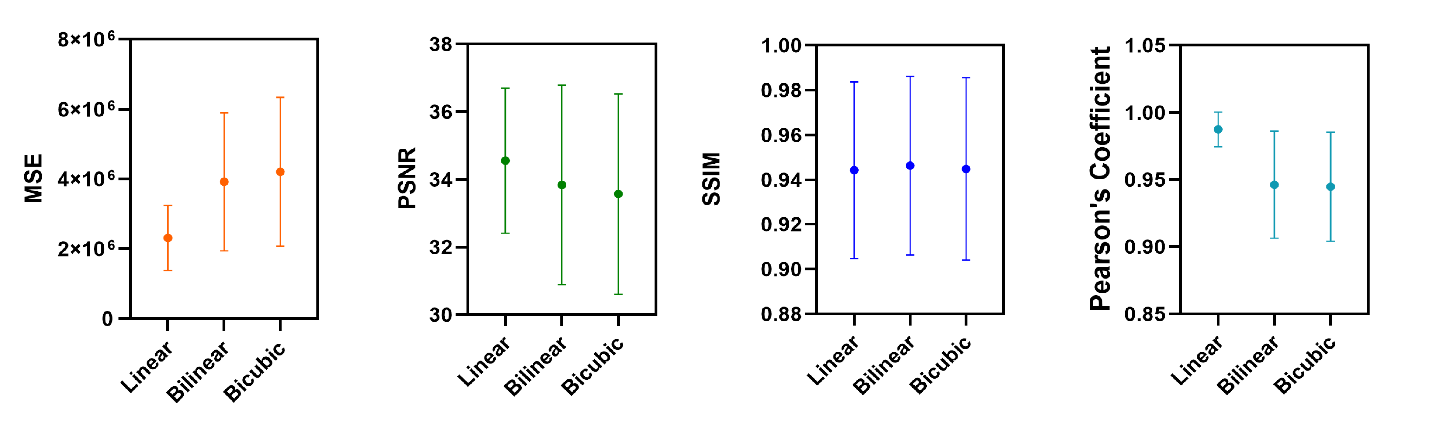


**Figure S3.** The comparison between the linear interpolation, bilinear interpolation, and bicubic interpolation. The metrics show similar performance between bilinear and bicubic interpolation (2D), while linear interpolation shows slightly better metrics, especially in MSE and PSNR. As a result, we used linear interpolation as a baseline to compare our models.


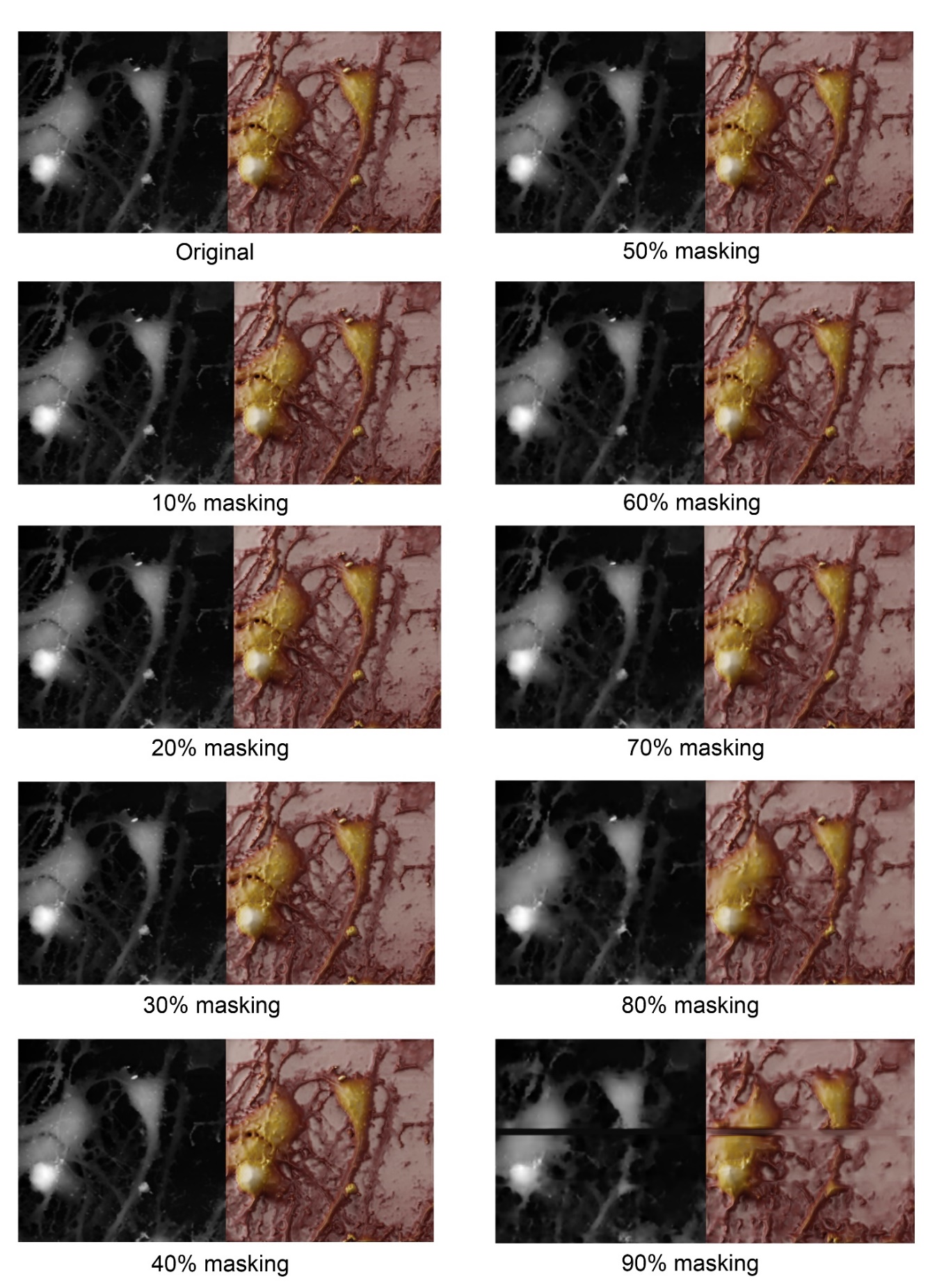


**Figure S4**. The effect of masking percentage on reconstruction quality using the Partial-CNN model is shown in grayscale (model’s raw output) and 3D RGB rendered images.


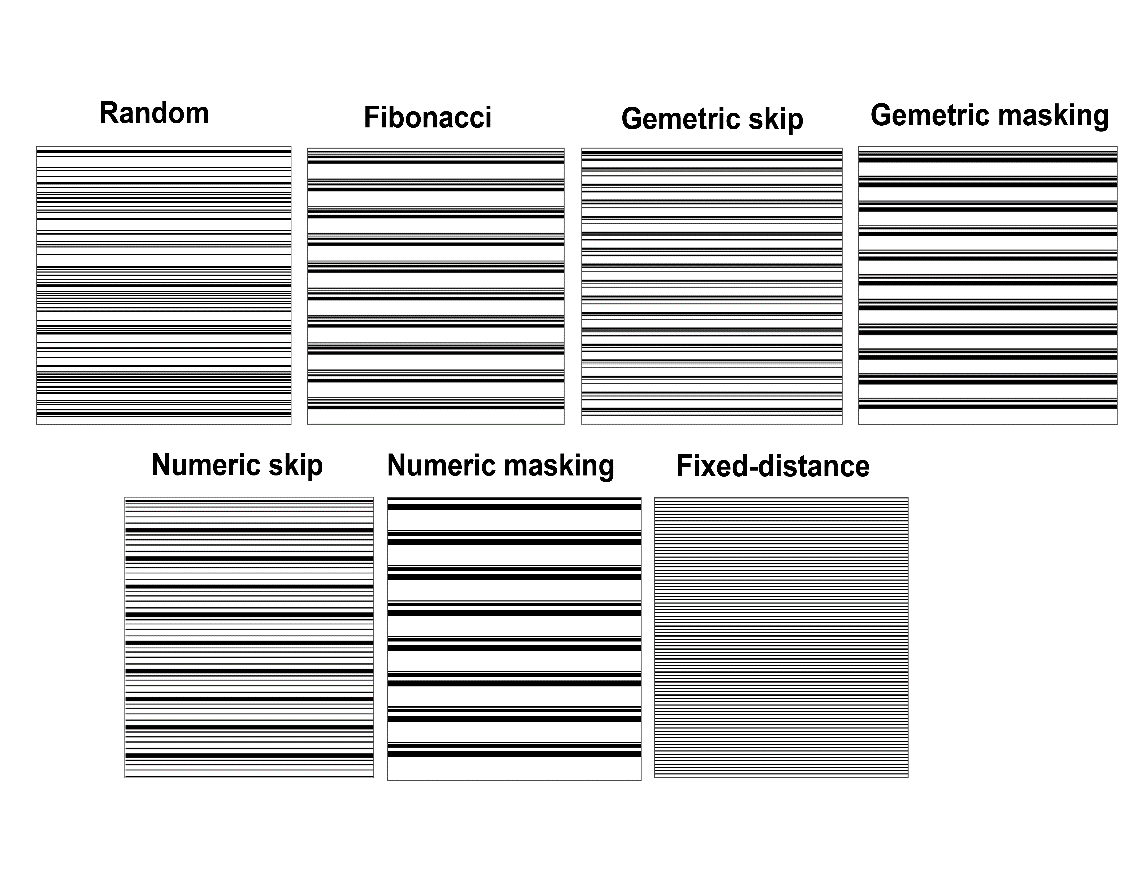
**Figure S5.** Example of random masking with excessive consecutive masked lines, leading to significant information loss.


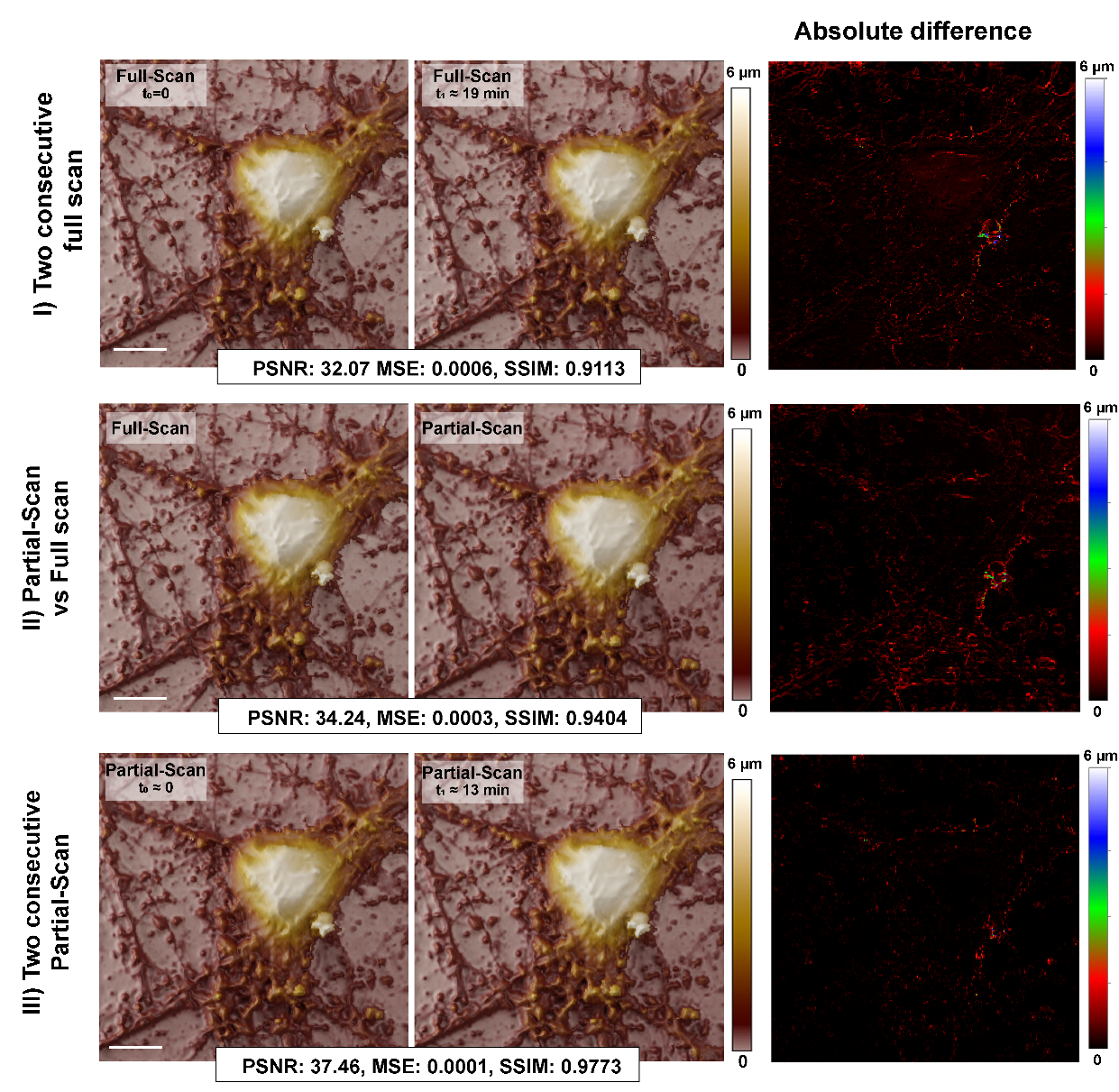
**Figure S6.** The comparison of absolute difference in I) two consecutive full scans, II) partial scan and full scan in the same area, and III) consecutive partial scans.

**Table S1**. Comparison of CNN and Partial-CNN models in the consumption of resources.

| Metrics | CNN | Partial CNN |
| --- | --- | --- |
| Trainable parameters* | 16,453,697 | 2,403,448 |
| Training time | 7,505.33 ± 489.98 | 3,376.33 ± 131.18 |
| GPU Utilization (%) | 64.54 ± 1.54 | 72.25 ± 0.42 |
| GPU Memory Usage (GB) | 32.76± 0.76 | 32.10 ± 1.69 |
| Total Energy Consumption (J) * | 49,754.33 ± 3013.84 | 20,105.62 ± 474.22 |

*Significant difference (P < 0.001)

**Table S2**. The controlled masking pattern strategies

| Masking Technique | Description | Series Formula | Pattern |
| --- | --- | --- | --- |
| Random | Random selection of horizontal lines with restriction of no masking more than 3 consecutive lines. |  |  |
| Fixed distance | Masking horizontal lines at regular intervals (Interval=3). | Interval = 3 | 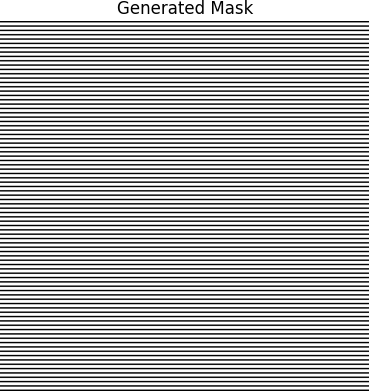 |
| Fibonacci | Masking lines based on the Fibonacci sequence, alternating between masking and skipping a Fibonacci number of lines. | $T_{n}= T_{n-1}- T_{n-2}$  Max. num = 3, repeat = 10 | 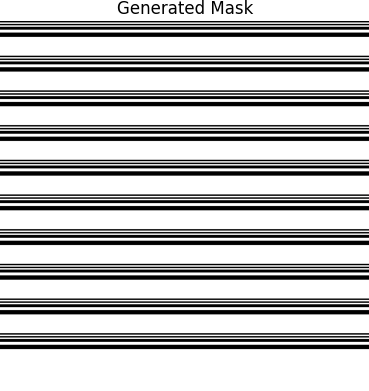 |
| Geometric mask | Masking lines using a geometric series to determine skips after masking each line. | $T_{n}=a.r^{(n-1)}$  a=1, r=2, Max. n = 3, repeat = 30 | 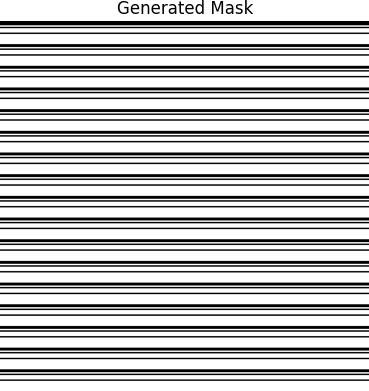 |
| Geometric skip | Masks and skips lines using a geometric progression with increasing gaps and masked lines. | $T_{n}=a.r^{(n-1)}$  a=1, r=2, Max. n = 3, repeat = 11 | 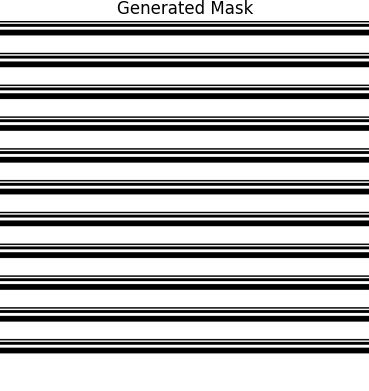 |
| Numeric mask | Masking lines using an arithmetic numeric series to determine skips after masking each line. | $T_{n}= a+\left( n-1 \right).d$  a=1, d=2, Max. num = 3, repeat = 10 | 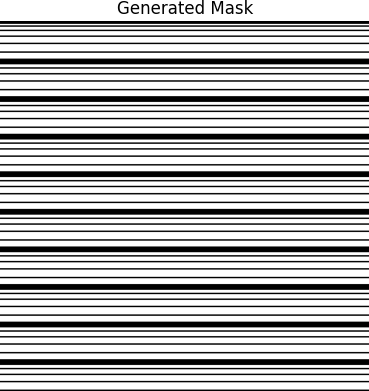 |
| Numeric skip | Masks and skips lines using a numeric progression, with increasing gaps and masked lines. | $T_{n}= a+\left( n-1 \right).d$  a=1, d=2, Max. num = 3, repeat = 8 | 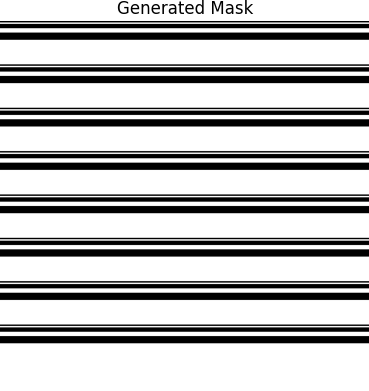 |

**Supporting Video S1**. The live time-lapse for more than 9 hours of neuroblastoma cells

See attached video
